# Bioinspired Topographic Surface Modification of Biomaterials

**DOI:** 10.3390/ma15072383

**Published:** 2022-03-24

**Authors:** Santiago Arango-Santander

**Affiliations:** GIOM Group, Faculty of Dentistry, Universidad Cooperativa de Colombia, Envigado 055422, Colombia; santiago.arango@campusucc.edu.co; Tel.: +57-604-4446065

**Keywords:** surface modification, biomimetics, sharklet, lotus leaf, bacterial adhesion, cell behavior

## Abstract

Physical surface modification is an approach that has been investigated over the last decade to reduce bacterial adhesion and improve cell attachment to biomaterials. Many techniques have been reported to modify surfaces, including the use of natural sources as inspiration to fabricate topographies on artificial surfaces. Biomimetics is a tool to take advantage of nature to solve human problems. Physical surface modification using animal and vegetal topographies as inspiration to reduce bacterial adhesion and improve cell attachment has been investigated in the last years, and the results have been very promising. However, just a few animal and plant surfaces have been used to modify the surface of biomaterials with these objectives, and only a small number of bacterial species and cell types have been tested. The purpose of this review is to present the most current results on topographic surface modification using animal and plant surfaces as inspiration to modify the surface of biomedical materials with the objective of reducing bacterial adhesion and improving cell behavior.

## 1. Introduction

Biomimetics, also known as biomimicry or bioinspiration, is a form of technology used by humans to improve our lives and solve some or our problems based on imitating nature [1]. The field of biomimetics has developed throughout history because humans have understood that nature is a vast source of inspiration to find solutions to many problems identified in many areas. Examples of areas that have benefited from biomimetics include industrial applications, such as the design and development of Velcro [2]; architecture to improve heating, cooling and ventilation systems based on termite nests [3]; engineering using models from different animals to design and improve aircraft [4], ships [5,6] and automobiles [7]; and medicine for a massive number of applications, including tissue engineering [8,9,10], cellular adhesion and biocompatibility [11] and reduction in bacterial adhesion [12,13].

Vast numbers of biomimetic approaches have been studied, including chemical and topographic surface modification of artificial materials following inspiration from natural surfaces. Regarding chemical surface modification, an array of nature-derived/inspired compounds has been used to modify the surface of biomaterials. For instance, silver nanoparticles (AgNPs) have been extensively investigated as a chemical surface modification material mainly due to its good stability and extensive antibacterial spectrum. However, they show some drawbacks, such as detachment, instability and cytotoxicity [14]. To counteract such disadvantages, polydopamine (PDA), a biopolymer inspired by mussels that has shown remarkable biocompatibility and adhesive properties, has been used to immobilize different antibacterial compounds [15], including AgNPs and antimicrobial peptides (AMP) [16], which have exhibited a remarkable antibacterial effect against species such as *Staphylococcus aureus*, *Escherichia coli* [17], *Streptococcus mutans* or *Porphyromonas gingivalis* [18]. Silicate nanoparticles have also been immobilized onto PDA to enhance osteogenesis of human mesenchymal stem cells [16]. Another example is self-assembled monolayers (SAMs), which are highly organized organic structures that allow to control different chemical properties of materials [19]. Using SAMs, some antibacterial coatings, created on different surfaces using bioinspired antimicrobial peptides [20], have shown effects against *S. aureus* and *E. coli* [21]. Incorporation of metal ions, such as silver or copper, onto SAMs has also exhibited bactericidal effects against a variety of bacterial species [19]. Furthermore, zinc oxide (ZnO) hierarchical structures synthesized using the *Cordia myxa* leaf showed high antibacterial activity against *E. coli* and *S. aureus* [22].

As for topographic modifications, natural surfaces have acquired immense biological topographic features at the micro and nano levels due to their prolonged evolution and adaptation. Bioinspired surfaces mimic such features to assist in improving the properties of artificial surfaces [23,24]. The most important surface attributes that are targeted by current investigations when bioinspired surfaces are used to reduce bacterial adhesion or improve cell attachment are roughness, wettability, surface energy and adhesion. Modification of these attributes using the topography from natural surfaces is advantageous since no chemical compounds are released into the environment where such surfaces are exerting their roles [25]. In the biomedical sciences, antibiotics have been the primary source to fight against bacterial colonization, but the indiscriminate use of such medications has led to the development of antimicrobial resistance (AMR), which is currently one of the most serious threats in medicine [26]. Topographic modification of the surface of biomaterials might assist in decreasing the use of antibiotics for treatment of implant-related infections, hence reducing the risk of AMR. An additional advantage of physical surface modification is that the well-known techniques used to modify the topography of biomaterials may reduce research expenses [27]. Topographic modification of artificial materials using natural surfaces as inspiration has been used in fields as diverse as marine applications to avoid fouling or reduce drag [24,28,29,30,31], preservation and safety of food products [32,33] or biomedical sciences [34,35,36,37,38,39], among many others.

When biomaterials are placed in biological environments, bacteria have the ability to adhere to their surfaces. They are adsorbed onto the surface by several means and then they aggregate and secrete extracellular matrix (ECM), which lead to irreversible adhesion to the surface and formation of a biofilm. Therefore, numerous strategies aiming at disrupting the interactions between bacteria and surfaces have been developed [40]. As mentioned, surfaces that have been topographically modified inactivate bacteria without the use of chemical compounds. Therefore, the destruction of bacterial species is governed by the interactions that occur at the interface between the bacterial cell and the topographic features from the modified surface, namely pillars, columns, rods, etc. The characteristics of those features, such as height, width, diameter and spacing, play a significant role in the response observed when bacteria come into contact with them. In addition, different bacterial species possess different characteristics, including different shapes, membrane configuration and composition and cell rigidity, which ultimately influence their own response to mechanical disruption [35,41].

Physically modified surfaces also exert a role when in contact with cells. Investigations have demonstrated that the interaction between surface topographies and different cell types influence cell morphology, behavior, alignment, migration and proliferation, among other characteristics [42,43,44], which eventually modify the interactions between cells and surfaces.

**Figure 1:**
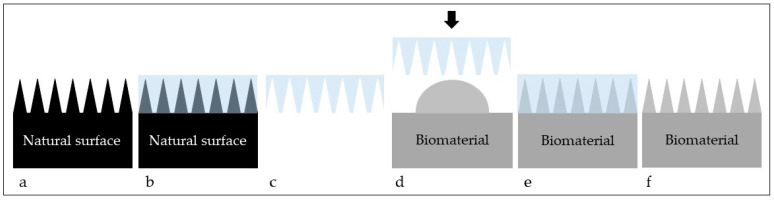
Schematics of the soft lithography process. A natural surface (**a**) is selected and its topography is duplicated using PDMS (**b**). The PDMS stamp that contains the negative impression of the topography is obtained (**c**). The PDMS stamp is used to transfer such topography to another surface (biomaterial) using different compounds, (i.e., silica, titanium dioxide, etc.) (**d**) and such compound is allowed to cure (**e**). Once the transferring process is complete, the PDMS stamp is removed and the topography from the natural surface will remain on the biomaterial’s surface (**f**).

The surface of a biomaterial may be topographically modified using different techniques, which belong to top-down or bottom-up methods. Top-down techniques, including direct laser interference lithography (DLIL) [45,46], nanoimprint lithography [47], photolithography [48], optical lithography, e-beam lithography, soft lithography (Figure 1) and scanning probe lithography, are based on creating structures with desired shapes and features starting from larger sizes and reducing them to the desired dimensions [49]. Bottom-up approaches, including atomic layer deposition [50], sol-gel and molecular self-assembly [51], rely on using atoms or small molecules as building blocks to fabricate multi-level structures [49]. Some of the mentioned techniques require the use of master models or templates, which are duplicated and transferred to the surface of artificial materials. Templates may be fabricated using photolithography [52] or other lithographic methods [53]. Through different topographic features found on the skin and surfaces of animals, insects and plants, nature may provide such templates.

The topography from different plants, animals and insects has been used as inspiration to modify the surface of artificial materials. Animal and insect surfaces have been the most extensively investigated, and the sharkskin has been one of the most studied animal sources [36,54,55,56,57], especially for its drag reduction, antibacterial and antifouling properties. Other animal surfaces have also been considered for different objectives, such as the strider´s leg [58] or butterfly´s wings [59] to increase surface hydrophobicity, the gecko´s feet to increase adhesion [60], the eagle owl´s feathers for noise reduction [61] and the wing of the cicada for antibacterial purposes [62].

**Figure 2:**
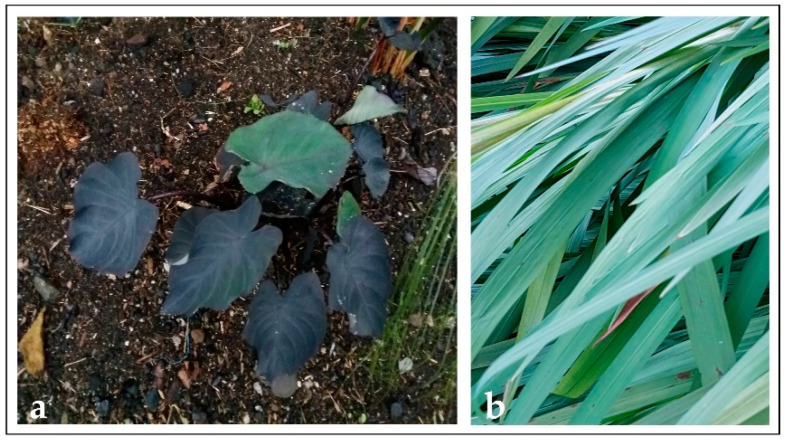
Leaves from (**a**) black taro (*Colocasia esculenta*) and (**b**) Montbretia (*Crocosmia aurea*).

Plants have also been proposed as models to modify the surface of different materials, although information on plants is scarcer. Some plant surfaces that have been reported in the literature include rice [63,64], lotus [65] and taro [66] leaves to increase surface hydrophobicity, rose petals for its anti-icing properties [67] or black taro, Montbretia (Figure 2) and giant Salvinia leaves to reduce bacterial adhesion [41]. The purpose of this review is to present the most relevant and current information on the use of animal, insect and plant surface topographies as inspiration to physically modify the surface of artificial biomedical materials with the objective of reducing microorganisms’ adhesion or improving cell adhesion and biocompatibility. Bioinspired chemical surface modification using compounds obtained from or based on natural sources to coat or otherwise transform the surface of artificial biomaterials, the techniques used to modify the surfaces and bioinspired physical surface modification in non-biomedical areas (textiles, food packaging, marine applications, oil-water separation, among many others) are beyond the scope of this paper as there are excellent reviews on those topics in the scientific literature.

## 2. Bioinspiration from Animal and Insect Surfaces

### 2.1. Topographic Features from Animal and Insect Models

The topography from the surface of animals and insects has been widely investigated to be used as surface models to modify the surface of artificial materials due to some interesting and advantageous properties, such as high hydrophobicity, roughness or surface features disposition. Many skins and external surfaces from different animals have been studied for different purposes. Each skin or external surface shows different features and distributions, the shape and size of such features is different among animals and insects, so these natural sources have been investigated and used depending on the final objective pursued by the modified synthetic surface.

**Figure 3:**
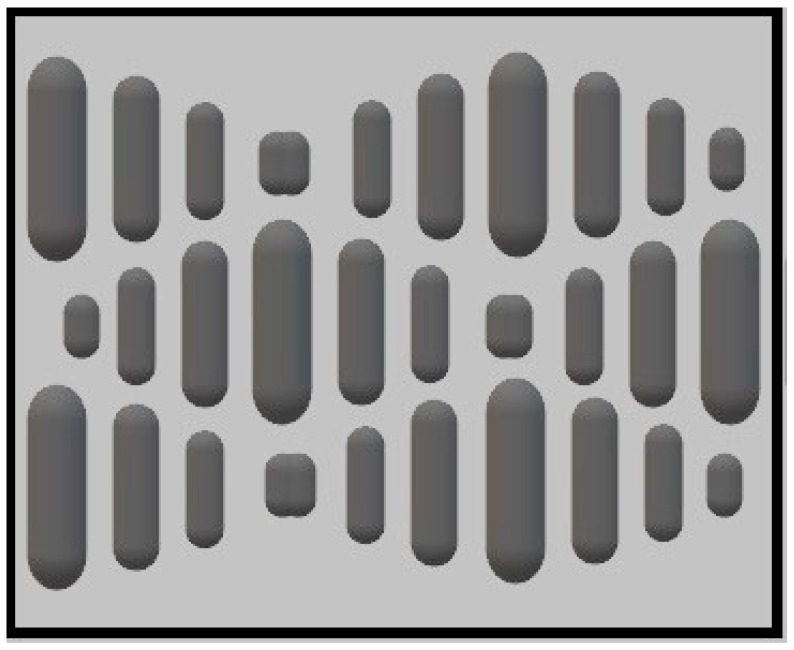
Schematics of the Sharklet pattern.

The most reported animal topography for modification of biomedical surfaces is the sharkskin, which has been studied to modify the surface of diverse materials due to its excellent self-cleaning and anti-fouling properties as a result of the microscopic shape and disposition of the denticles (diamond-shaped scales covering the outer surface of this animal) [68]. As a result, a model from such skin, known as Sharklet, has been developed. This topography consists of rectangular features with different lengths (4–16 µm), a width of 2 µm and a height of 3 µm disposed in a diamond-shaped periodic array at a fixed spacing of 2 μm between adjacent features (Figure 3) [56].

The topography from the cicada wing has also been proposed to modify the surface of artificial materials. The outer surface is composed of two sublayers, known as inner and outer epicuticles, which contain proteins and lipids (inner) and polymerized hydrocarbons (outer). Other chemical constituents, including fatty acids, sterols, alcohols and esters, have been found in different species of cicada [69]. These epicuticles feature a highly ordered array of nanopillars that exhibit different height, width and pitch values among different cicada species [70]. For instance, the *Psaltoda claripennis* cicada wing shows hexagonal arrays of conical nanopillars in the range of 200 nm in height, 100 nm in diameter at the base and 60 nm at the tip and spaced around 170 nm from center to center [71]. However, other authors suggest that this morphology is more closely comparable to nanocones, since the base of the structure is wider than the tip [72,73].

The dragonfly wing (*Diplacodes bipunctata*) has also been studied. Ivanova et al. [74] found nanopillars having hierarchical features that tend to form a network at the base, while the tips remain disconnected or form tip clusters. The size and shape of the clusters are random and show a sigmoidal population distribution below 90 nm, some exhibiting under 30 nm in diameter. The clusters show a spatial distribution between 200 and 1.800 nm in diameter. Nguyen et al. [75] chemically characterized the wing from *Hemicordulia tau* and found that the nanoscale pillars present at the epicuticle are composed mainly of aliphatic hydrocarbons and palmitic acid.

The gecko skin has been tested for many applications, including its antibacterial capabilities. Watson et al. [76] characterized the abdominal and posterior skin of the *Lucasium steindachneri* species. They found dome-shaped pigmented scales arranged in a hexagonal patterning. The scales from the skin from the back of the animal exhibited 100–190 µm in diameter and around 50 µm in height. In the abdominal area, larger scales with more spacing were found. Hairs (spinules) of up to 4 µm in length, with sub-micron spacing and a small radius of curvature typically from 10 to 20 nm, were also found. Β-keratin and lipids are the main components of the surface of the gecko skin [77].

The topography of a planthopper (*Desudaba danae*) has been characterized due to its non-wetting behavior and self-cleaning properties. The hindwing consists of micro asperities in the range of 6 µm in height, 500 nm in length, 45 to 50 nm in diameter and a spatial separation of 14 µm on average. The forewing does not show this structure, but a series of grouped structures exhibiting different roughness dimensions [78]. A summary of the topographic features and applications of animal and insect surfaces is presented in Table 1.

### 2.2. Microorganisms Adhesion and Colonization

Animal and insect surfaces have served as inspiration to be used in many areas, but the field of using topographically modified bioinspired surfaces to reduce bacterial adhesion to biomaterials has received less attention than other areas, such as marine biofouling. However, this area is growing and more information has been made available in the scientific literature within the last years.

Several investigations have made use of the Sharklet topography for surface modification of various biomaterials. Chung et al. [36] modified the surface of a poly (dimethyl siloxane, PDMS) elastomer and assessed the adhesion and colonization of *S. aureus*. They observed a reduction in colonization and surface coverage, even after 21 days, when compared the modified PDMS surface with a smooth one. Mann et al. [84] modified the surface of an acrylic film and compared the adhesion of methicillin-sensitive *S. aureus* and methicillin-resistant *S. aureus* to modified versus smooth surfaces and found reductions of 99% and 98%, respectively, in the adhesion of these bacterial species to modified surfaces. Mann et al. [85] applied the Sharklet model to modify the surface of a thermoplastic polyurethane material employed in the fabrication of endotracheal tubes. Then, they assessed the adhesion of *Pseudomonas aeruginosa* and methicillin-resistant *S. aureus* to such modified surface and compared it with a smooth surface. They found a reduction of over 70% in the adhesion of the investigated species and better airflow through the lumen of the endotracheal tube since there was lower accumulation of mucus on the surface due to a reduction in bacterial colonization. Reddy et al. [86] studied how the physical modification of a silicone elastomer using Sharklet affected the adhesion and colonization of *E. coli*. Their results showed that modified surfaces significantly reduced (>47%) the adhesion and colonization of such pathogen when compared with smooth surfaces of the same material. May et al. [37] also used it to modify the surface of a polymer employed for endotracheal tubes and evaluated the adhesion and biofilm formation of five pathogens responsible for ventilator-associated pneumonia (VAP) (methicillin-resistant *S. aureus*, *Pseudomonas aeruginosa*, *Klebsiella pneumonia*, *Acinetobacter baumannii*, and *E. coli*) and found a significant reduction (from 95.6% up to 99.9%) in the adhesion and colonization of these pathogens to this polymer. Following the same line, May et al. [87] used this topography to modify a thermoplastic polyurethane, used as a catheter material, and evaluated the adhesion and colonization of *S. aureus* and *Staphylococcus epidermidis*. They found significant reductions (70% and 71%, respectively) in the colonization to modified versus unmodified surfaces. Arisoy et al. [88] used the same physical surface modification approach with Sharklet to modify the surface of poly(ethylene terephthalate, PET), but added a coating of TiO_2_ nanoparticles at different concentrations (0, 10 and 50 wt%) to further increase the antibacterial effect against *S. aureus* and *E. coli* due to the photocatalytic effect exhibited by TiO_2_. In general, they found a significant reduction in the coverage of *E. coli* to the patterned surfaces (70–85%) and 85–95% reduction in *S. aureus* adhesion. Interestingly, there was no significant differences between the antifouling activity of the patterned surfaces with or without TiO_2,_ which indicates that surface topography was more important than the chemical composition of the surfaces in terms of reducing bacterial adhesion of these species. Liu et al. [68] evaluated the modification of polypropylene and silicone surfaces to evaluate whether a reduction in *S. aureus*, *E. coli*, bacteriophage T4, influenza B virus and human coronavirus colonization could be observed. Their findings showed substantial in vitro reductions (ranging from 63.5% to 97.8%) in colonization of these pathogens on such surfaces. Rostami et al. [89] fabricated a chitosan membrane based on the sharkskin and chemically modified it with graphene oxide to assess the synergistic effect of topographic and chemical surface modifications against *S. aureus* and *E. coli*. They found reductions of over 70% in the adhesion of both bacterial strains.

While these investigations were performed in vitro, Magyar et al. [80] compared the bacterial adhesion to modified versus smooth silicone urinary catheters in 50 male patients who required temporary urethral catheterization from 3 to 30 days in their phase I randomized open label interventional trial and found a significant reduction in biofilm formation on the surface of the modified catheters.

Regarding the use of the topography from cicada wings, Kelleher et al. [62] studied how the topography from three species (*Megapomponia intermedia*, *Ayuthia spectabile* and *Cryptotympana aguila*) affected the colonization of *P. fluorescens* and found a reduction between 75% and 80%. Dahghani et al. [90] obtained similar results when assessed the adhesion of *P. aeruginosa* to the surface of the wings from *Psalmocharias genus*, *Psalmocharias querula* and *Psalmocharias akesensis.* Even though these authors did not modify any artificial surface and performed their experiments directly on the cicadae wings, their results open the possibility of using such wings as models to modify biomaterials and confirm whether their findings could be achieved on biomimetically modified artificial surfaces. Shahali et al. [81] assessed the topography of the wings from other cicada species (*Psaltoda claripennis*, *Aleeta curvicosta* and *Palapsalta eyrei*) and evaluated the anti-bacterial properties of such topographies on the adhesion of *P. aeruginosa* y *S. aureus*. They found a reduction in the adhesion of such bacterial species directly to the wings of the three cicadae. In addition, the authors used electron beam lithography to transfer the topography from the different wings to titanium surfaces and obtained similar results regarding reduction in bacterial adhesion. Hazell et al. [82] fabricated nanocone arrays of different aspect ratios on the surface of PET mimicking the cicada wing topography and tested the bactericidal effect on *E. coli* and *K. pneumoniae.* They found statistically significant differences in the bactericidal effect when compared with a smooth PET surface.

Comparable results were obtained by Watson et al. [78], who investigated the antimicrobial effect of the cuticle from the wing of the plant hopper *Desudaba danae* on the adhesion and colonization of *P. gingivalis*. Even though they assessed the effect directly on the wing of the insect, their conclusions include the potential of such surfaces to be used in biomimetically-modified synthetic surfaces and biomaterials to reduce the adhesion of different microorganisms. Bhadra et al. [91] used a hydrothermal process to create nanoarrays on the surface of commercially pure grade-2 titanium surfaces mimicking the surface of the dragonfly. They exposed the modified surfaces to *P. aeruginosa* and *S. aureus* to evaluate their antibacterial effect and found that 50% of *P*. *aeruginosa* and 20% of *S. aureus* cells were eliminated after being in contact with the surface.

### 2.3. Cellular Adhesion and Biocompatibility

The topic of using biomimetic surfaces transferred from animals or insects to evaluate the behavior of cells has not been as extensively reported in the literature. As already mentioned, many investigations have demonstrated that micro and nano topographies influence the behavior of cells, but such topographic features have been carefully fabricated using different techniques to control the size, height, spacing and other characteristics of the pillars, cones, columns or other shapes used to artificially create topographies. Watson et al. [76] evaluated whether the surface of the gecko skin could be harmful to human dental pulp stem cells (hDPSCs). They found that the gecko skin showed compatibility with the hDPSCs and cell growth and proliferation occurred. Similar results were obtained by Watson et al. [78] when they assessed the biocompatibility between the planthopper wing and two cell lines (human dental fibroblasts and SHED-MSCs) and found compatibility for attachment, division and growth.

However, the above-mentioned papers investigated the effect on the natural surfaces without modifying an artificial biomaterial. Magin et al. [83] modified the surface of PDMS using the Sharklet model to evaluate the behavior of lens epithelial cells (LEC) when in contact with such modified surface versus an unmodified surface. They found a reduction in LEC coverage of 80%, which, in turn, demonstrated a reduction in posterior capsular opacification (PCO). Li et al. [92] used laser surface texturing to modify the surface of Ti6Al4V samples following the topography of the toe pads from the tree frog. They observed high proliferation and viability of mouse calvaria osteoblasts (MC3T3-E1) in contact with such hierarchically modified surfaces. Bhadra et al. [91] also subjected primary human fibroblasts (pHF) to the presence of the aforementioned titanium surface mimicking the dragonfly for up to 10 days and compared the cell behavior with a smooth titanium surface. After 10 days, they observed that the pHF had adhered, proliferated, aligned and formed multiple layers of cells on the nanostructured surface. In addition, they exhibited an extended morphology. On the unmodified titanium surface, cells distributed more evenly, conserved their shape and formed a monolayer. Mobini et al. [93] found that the sharklet topography promoted the alignment and attachment of Schwann cells, while inhibited fibroblasts. They observed that Schwann cells extensions were stretched out and adhered to the top and edge of the sharklet features and their morphology was elongated within the microchannels, while fibroblasts were flattened and their cytoplasm was expanded over and between the sharklet and microchannels. Rostami et al. [89] also assessed the biocompatibility of graphene oxide-sharkskin modified chitosan membranes and found increased cytocompatibility between modified surfaces and human keratinocytes (HaCaT) and mouse fibroblast (L929) cell lines. More investigations are needed to observe how different cell types adapt to biomimetically-modified surfaces in order to understand the underlying mechanisms of attachment and spreading to develop tailor-made surfaces that improve the behavior of cells in contact with these surfaces. These results demonstrated that eukaryotic cells adapt much better than prokaryotic cells to topographic surface features created or otherwise present on the surface of materials used for biomedical applications.

## 3. Bioinspiration from Vegetal Surfaces

### 3.1. Topographic Features from Vegetal Models

**Figure 4:**
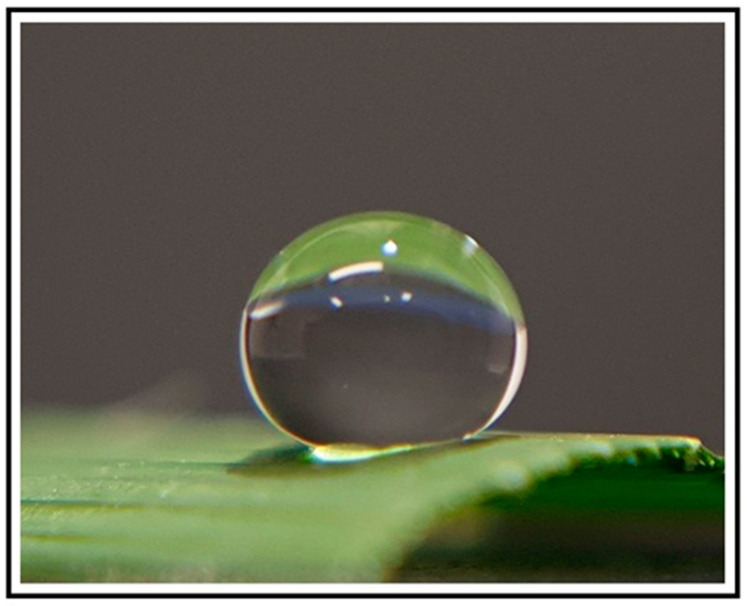
High hydrophobicity exhibited by a vegetal material (*C. aurea*).

**Figure 5:**
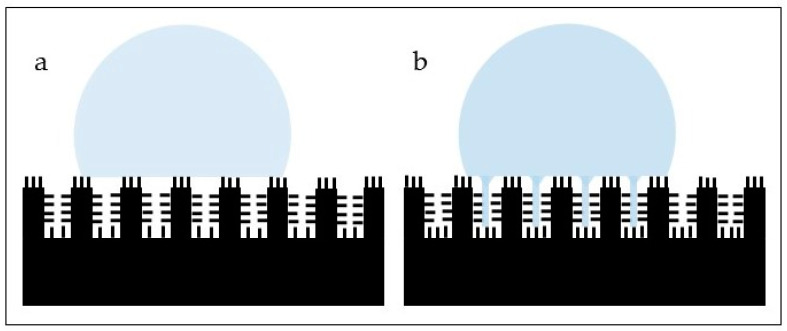
Lotus (**a**) and rose petal (**b**) effects.

Plant and vegetal sources have been investigated to modify the surface of biomaterials due mostly to their high hydrophobicity (Figure 3) and self-cleaning properties. However, information on using the topography from plants and leaves to modify the surface of biomaterials in the scientific literature is scarce. As the sharkskin has been the most investigated model in animal biomimetics, the lotus leaf has been the most addressed when looking for inspiration from vegetal sources. The lotus (*Nelumbo nucifera*) leaf has a hierarchical surface characterized by protrusions and valleys ranging from 3–10 µm. The protrusions possess nanometric particles (70–100 nm in size) of a hydrophobic wax-like material. This wax material is mainly composed by nonacosanediols and nonacosan-10-ol on the upper side of the leaf (65% and 22%, respectively) and by nonacosan-10-ol, diols and alkanes on the underside of the leaf (53%, 15% and 18%, respectively) [94]. The subsurface layer shows nano sticks with diameters around 50 nm randomly distributed [95]. The most relevant characteristics are its high hydrophobicity (Figure 4) and self-cleaning (“lotus leaf effect”, Figure 5a) abilities, in which water droplets roll off easily from the surface [96,97]. These properties have been associated with many effects, including antibacterial.

Rose petals have hierarchical structures with micro-papillae of around 20 µm in diameter and nanometric cuticular folds of around 730 nm in width [98,99]. Such hierarchical surface is responsible for the “rose petal effect”, in which water droplets are highly adhered to the superhydrophobic surface of the petal (Figure 5b) [96,97]. Chemical analysis from *Rosa rugosa* show that the petals are composed by phenolic acids, tannins, flavonoids, carotenoids and polysaccharides [99]. Rice leaves show papillae around 5–8 µm in height on the surface, which are arranged in one-dimensional parallel order. The sublayer shows nanometric pins proportionally distributed to enhance the amount of air trapped in the surface [93]. Taro leaves show elliptic protrusions with diameters of around 10 µm uniformly distributed in nest-like caves and nanometric pins disseminated on the surface, resulting in a hierarchical structure [93]. The chemical composition of taro leaves includes the presence of phenolic acids, flavonoids, saponins, tannins and alkaloids [100]. The *S. molesta* leaf is covered by hairs capped with a crown-like structure on the upper side. Each hair is composed by a 1.5 mm-long stalk and the tip exhibits four rounded filaments connected at the apex, which form a crown-like structure of about 500 µm in height [101]. Table 2 summarizes the topographic features of some vegetal materials that have been used to topographically modify the surface of biomaterials.

### 3.2. Microorganisms Adhesion and Colonization

Jian et al. [102] tested anti-fouling and bactericidal activities directly on the lotus leaf. They also modified the surface of silicon wafers at the micro and nano scales to mimic the hierarchical structure of the leaf and found drastic reductions of over 99% in the adhesion and colonization of *E. coli* for periods ranging from 3 to 24 h. The topography of rose petals has also been proposed to modify the surface of materials. Cao et al. [98] used PDMS to duplicate the topography of the rose petal and then transferred it to an epoxy surface. They assessed the antibacterial capability of such topography against *S. epidermidis* and *P. aeruginosa* and found a reduction of over 86% in the adhesion of both bacterial species to the modified surfaces.

Other plants and leaves have also been tested. Bixler et al. [64] assessed the effect of the rice leaves topography on the adhesion and biofilm formation of *E. coli* using different procedures to modify the surface of PDMS and found different values of reduction related to the different methodologies used in their work.

**Figure 6:**
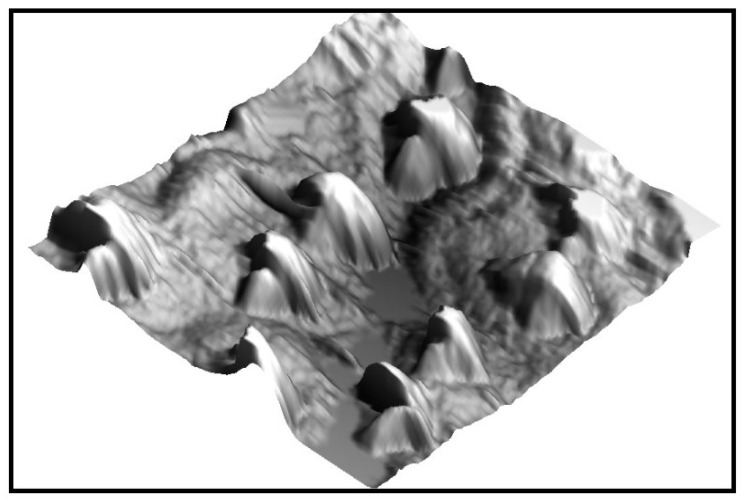
AFM image of the surface of *C. aurea*.

Our previous works tested the antibacterial effect shown by the topography of black taro (*Colocasia esculenta*), giant Salvinia (*Salvinia molesta*) and Montbretia (*Crocosmia aurea*, Figure 6) against *S. mutans*. The topographies from these leaves were duplicated using PDMS and stainless steel and titanium alloys surfaces, used for orthodontic purposes, were modified. The results showed an important reduction in bacterial adhesion to such surfaces, except the stainless-steel surface that was modified using the *S. molesta* topography, which showed an increase in adhesion [38,39].

### 3.3. Cellular Adhesion and Biocompatibility

The field of cellular adhesion and biocompatibility of modified surfaces using inspiration from vegetal sources is even more unexplored. Öztürk-Öncel et al. [103] modified the surface of PDMS using the topography from red and white rose petals. Then, they functionalized the modified surfaces with type 4 collagen and hyaluronic acid and subjected them to bovine corneal endothelial cells (CECs). They observed proliferation and viability of up to 7 days of these cells in contact with modified surfaces functionalized with collagen, but not with hyaluronic acid. Ramaswamy et al. [104] modified the surface of hydroxyapatite (HAp) using the topography from three leaves (parsley—*Petroselinum crispum*, rose—*Rosa kordesii* and daisy—*Orchidaceae*). Thus, they obtained three bioinspired patterns, namely honeycomb, pillars and isolated islands based on the topographies from the natural leaves. Then, they placed human adipose-derived stem cells (ADSCs) in contact with such surfaces and observed their behavior. They found flattening and elongated morphology and reduced cell protrusions.

## 4. Mechanisms Involved in Reduction in Bacterial Adhesion and Improvement of Cell Attachment

### 4.1. Reduction in Bacterial Adhesion and Bactericidal Mechanisms

A common conclusion among investigations using animal or insect bioinspired topographies is that a reduction in the adhesion and colonization of diverse bacterial species to different materials is obtained, which is a very promising approach to work synergistically with other methods to control the amount of bacterial biofilm on the surface of biomedical materials. However, the exact mechanisms as to why these surfaces reduce the adhesion and colonization of bacteria remain to be fully elucidated. It has been hypothesized that the presence of the sharkskin surface features disrupts the biofilm uniformity, leading to reduction in bacterial adhesion and biofilm coverage [87]. Another hypothesis proposed that bacteria pattern spontaneously on a tridimensional arrangement because bacteria align according to the topographic features on the surface. This depends on the size and spacing of such features, and this alignment changes as the spacing approaches the size of a bacterium [35]. It is important to consider that this conclusion was drawn after using arrays that were carefully constructed and their dimensions were judiciously controlled, which cannot always be guaranteed, especially when real natural surfaces are duplicated and transferred to otherwise smooth biomaterials. Mandal et al. [105] observed that bacteria do not form colonies on nanostructured surfaces possibly owing to the incapability of bacterial cells to divide and grow due to the presence of nanometric features, which might act as obstacles. Other hypotheses that attempt to explain such reduction include the presence of air pockets that remain between topographic features and hinder bacterial adhesion [106]; the non-wetting nature of the topography, related to the air cushions, that make the surface unavailable for bacteria [37,74,78] or bacterial membrane stretching or puncture as a result of the contact between the bacterium and the features, especially when the latter display high-aspect ratio and sharp shapes. Xue et al. [107] developed a theoretical mechanical model to attempt to explain the antibacterial effect shown by nano structures such as the nano pillars found on the cicada wings. According to this model, gravity and nonspecific forces, such as van der Waals, play a role in cell destruction by rupture, which render Gram-negative bacteria more susceptible to nanoscale features. They also concluded that the geometric parameters of the surface features determine the bactericidal nature of such a surface. Velic et al. [108] performed a three-dimensional finite element simulation to understand whether the bacterial envelope gets ruptured when bacteria are located in between protruding pillars. Instead, they found that the rupturing mechanism is more related to envelope strain and rupture takes place predominantly at the tip of the pillar. This work also demonstrated an increase in envelope deformation when bacteria adhered to nanopatterns with small radii and spacing among features. Nonetheless, the contact between bacteria and nano features may eventually lead to cell death [75,87,88]. The work by Jenkins et al. [109], using Gram-negative (*E. coli* and *Klebsiella pneumoniae*) and Gram-positive (*S. aureus*) bacteria, analyzed more in-depth the mechanist processes associated with the destruction of bacterial cells by nanopillars. The bacterial species were placed in contact with TiO_2_ nanopillars, mimicked from the dragonfly skin, to observe the behavior of such cells. As expected, due to the thickness of the cell wall, they found that Gram-negative bacteria were more susceptible to deformation and puncture by the nanopillars, but no cell lysis was observed. Gram-positive species tested showed better resistance to membrane deformation and rupture, although some deformation was also observed, but no lysis was found. The authors observed that production of reactive oxygen species (ROS) increased and higher levels of H_2_O_2_ were found in the nanopatterned surface versus the control smooth surface. This oxidative stress may impair some basic functions, such as bacterial growth and biofilm formation. In addition, this investigation showed that nanopillars induced cell impedance, which may reduce the capacity of bacteria to replicate on nanostructured surfaces. Membrane rupture and cell destruction due to the presence of nanopillar was not the predominant mechanism observed in this work.

The observed reductions in microorganisms’ adhesion and colonization, however, must be carefully analyzed since the high diversity of shapes and topographies provided by natural sources, as well as the differences between bacterial species (i.e., Gram-positive vs. Gram-negative, rods vs. cocci, etc.), make the responses highly variable and some topographies have provided better results with specific bacterial groups, but not with other species [74]. Moreover, some natural patterns have shown little or no effect at all on determined bacterial species [38]. Therefore, the mechanistic basis of reduction in bacterial adhesion and killing with modified structured surfaces is multifactorial [103] and must be further elucidated.

### 4.2. Mechanisms Related to Enhancemente in Cell Attachment

Cells respond differently to patterns because they have the ability to change their morphology depending on the environment where they are, unlike bacteria. Consequently, different patterns elicit diverse responses in different cell types. Most investigations regarding the behavior of different cell types when in contact with modified surfaces have been performed on surfaces where fabrication of micro and nano topographies is carefully controlled [44,110,111,112,113]. In a structure composed of micro and nano poly(L-lactide, PLLA) features, fibroblasts and osteoblasts responded preferably to the hierarchical structures instead of a smooth surface. The fabrication of these structures did not follow a biomimetic approach as neither an animal surface nor a vegetal one was used as inspiration to fabricate the patterns, but this work shows the preference of this cell types for hierarchical structures [114]. Likewise, Raczkowska et al. [115] fabricated poly(cholesteryl methacylate, PChMa) coatings composed of PChMa brushes and tested the biocompatibility of these structured coatings against granulosa and non-malignant bladder cancer (HCV29 line) cells. As mentioned, these cell types showed a predilection for the structured glass surface vs. the smooth one, even though the inspiration to fabricate the brushes was not based on animal or plant surfaces. Liu et al. [116] evaluated the response of the nucleus of mesenchymal stem cells (MSC) to the presence of surface features (micropillars) made of poly(lactide-co-glycolide, PLGA). They observed that this nucleus suffered severe deformation, followed by a partial recovery.

These investigations have demonstrated that eukaryotic cells adapt better to patterned surfaces than prokaryotic cells. However, this behavior seems to be associated with cell-related aspects, such as the cell´s type [117], origin, size and function. Some cell types seem to interact with the top of the features while others prefer the inter-feature spacing or the flat surface between features [44]. Therefore, the exact mechanisms leading to the response of eukaryotic cells to the presence of surface features remain unclear.

## 5. Conclusions, Challenges and Future Prospects

The field of topographic surface modification of biomedical materials using inspiration from nature has evolved in the last years due to the promising results obtained in numerous in vitro investigations. Topographic surface modification of biomaterials inspired by natural sources has demonstrated, so far, that it is a tool worth investigating when considering non-chemical alternatives to improve the performance of artificial biomedical surfaces. Nature offers an immense array of surfaces and topographies that may be used to modify the surface of synthetic biomaterials to improve their behavior when in contact with bacteria, fungi or cells, which will ultimately improve their performance within the biological environments where they will be used. Results have shown that the response of bacteria to topographic features is highly variable, so the mechanisms must be really understood in order to start fabricating arrays based on natural surfaces that have a much stronger effect on different bacterial species. Future works should address other natural topographies and their correlation with bacterial and cell adhesion. In addition, multi-species investigations should be performed. Surface patterning must be fine-tuned in order to elicit positive responses from bacteria (reduction) and cells (enhancement). Moreover, investigations of artificial biomaterials modified using topographies from natural sources should aim at clinical evaluation to develop biomaterials that can be used in real scenarios. It is imperative to find suitable alternatives to chemical surface modification and, especially, the use of antibiotics. The encouraging in vitro results must lead the way into more in vivo experiments and clinical trials, as well as characterization of more animal and vegetal surfaces that show properties similar to those already investigated. Immense possibilities are open to continue investigating more natural sources and their interactions with different microorganisms and cell types to fully elucidate the mechanisms behind the remarkable results that have been observed and to take advantage of all the possibilities that nature has to offer to improve the behavior of biomaterials.

## Figures and Tables

**Table 1:** Topographic features and biomedical applications of different animal and insect surfaces.

Animal/Insect	Topography	Applications in Biomaterials	References
Sharkskin	Denticles: scales of diamond-shape with a raised ridge and concave groove that show some nanostructures. The Sharklet model is made of rectangular features of 4–16 µm in length, around 2 µm of width and a height of 3 µm at a spacing of around 2 μm between adjacent features.	Reduction in bacterial adhesion alone or coupled with other chemical and photocatalytic compounds	[79,80]
Cicada wings	Highly ordered array of nanopillars or nanocones of different sizes, heights and spatial distribution depending on the species.	Antibacterial	[62,81,82]
Dragonfly skin (*Diplacodes bipunctata*)	Nanopillar clusters of random size, height and spacing	Antibacterial	[74]
Gecko skin (*Lucasium steindachneri*)	Dome-shaped pigmented scales arranged in a hexagonal patterning. Scales from 100–190 µm in diameter and around 50 µm in height at the back, larger scales with more spacing in the abdominal area. Spinules (hairs) up to 4 µm in length, with sub-micron spacing and a small radius of curvature typically from 10 to 20 nm.	Antibacterial	[76]
Planthopper wing (*Desudaba danae*)	Hindwing: micro asperities of around 6 µm in height, 500 nm in length, 45–50 nm in diameter at a spacing of around 14 µm. Forewing: grouped structures of various roughness dimensions.	Antibacterial Cell compatibility	[78]
Butterfly wing (*Morpho aega*)	The wing is covered with micro scales, parallel ridges and tile-like microstructures, nanoscale ribs and lamella-stacking nano-stripe structures	Easy cleaning coatings	[79]
Tree frog toe pad (*Litoria caerulea*)	Peg-studded hexagonal cells separated by channels and by finer pegs on the flattened surface of each hexagonal cell	Enhanced attachment	[83]

**Table 2:** Topographic features and biomedical applications of different vegetal surfaces.

Vegetal	Topography	Applications in Biomaterials	References
Lotus leaf (*Nelumbo nucifera*)	Hierarchical surface with protrusions and valleys ranging from 3–10 µm. Nanometric particles (70–100 nm in size) of a hydrophobic wax-like material in the protrusions. Subsurface layer has nano sticks with diameters around 50 nm randomly distributed	Reduction in bacterial adhesion Antibacterial	[95,96,97]
Rice leaves	Papillae around 5–8 µm in height on the surface arranged in one-dimensional parallel order. Sublayer shows nanometric pins proportionally distributed	Reduction in bacterial adhesion	[95]
Rose petals	Hierarchical structures with micro-papillae of around 20 µm in diameter. Nanometric cuticular folds of around 730 nm in width	Reduction in bacterial adhesion Cell attachment	[95]
Taro leaves (*Colocasia esculenta*)	Hierarchical structure with elliptic protrusions with diameters of around 10 µm uniformly distributed in nest-like caves. Nanometric pins disseminated on the surface	Reduction in bacterial adhesion	[95]

## Data Availability

Not applicable.
